# Social Determinants of Health Factors for Gene–Environment COVID‐19 Research: Challenges and Opportunities

**DOI:** 10.1002/ggn2.202100056

**Published:** 2022-03-09

**Authors:** Jimmy Phuong, Naomi O. Riches, Charisse Madlock‐Brown, Deborah Duran, Luca Calzoni, Juan C. Espinoza, Gora Datta, Ramakanth Kavuluru, Nicole G. Weiskopf, Cavin K. Ward‐Caviness, Asiyah Yu Lin

**Affiliations:** ^1^ Division of Biomedical and Health Informatics University of Washington Seattle WA 98195 USA; ^2^ Harborview Injury Prevention Research Center University of Washington Seattle WA 98104 USA; ^3^ Department of Biomedical Informatics University of Utah School of Medicine Salt Lake City UT 84108‐3514 USA; ^4^ Health Informatics and Information Management University of Tennessee Health Science Center Memphis TN 38163 USA; ^5^ National Institute on Minority Health and Health Disparities (NIMHD) National Institutes of Health Bethesda MD 20892‐5465 USA; ^6^ Department of Biomedical Informatics University of Pittsburgh Pittsburgh PA 15206 USA; ^7^ Department of Pediatrics Children's Hospital Los Angeles Los Angeles CA 90015 USA; ^8^ Department of Civil and Environmental Engineering University of California at Berkeley Berkeley CA 94720 USA; ^9^ Division of Biomedical Informatics Department of Internal Medicine University of Kentucky Lexington KY 40506 USA; ^10^ Department of Medical Informatics & Clinical Epidemiology Oregon Health & Science University Portland OR 97239 USA; ^11^ Center for Public Health and Environmental Assessment US Environmental Protection Agency Chapel Hill NC 27514 USA; ^12^ National Human Genome Research Institute (NHGRI) National Institutes of Health Bethesda MD 20892‐2152 USA

**Keywords:** COVID‐19, environmental factors, genomics, GxE, medical informatics, social determinant of health

## Abstract

The characteristics of a person's health status are often guided by how they live, grow, learn, their genetics, as well as their access to health care. Yet, all too often, studies examining the relationship between social determinants of health (behavioral, sociocultural, and physical environmental factors), the role of demographics, and health outcomes poorly represent these relationships, leading to misinterpretations, limited study reproducibility, and datasets with limited representativeness and secondary research use capacity. This is a profound hurdle in what questions can or cannot be rigorously studied about COVID‐19. In practice, gene–environment interactions studies have paved the way for including these factors into research. Similarly, our understanding of social determinants of health continues to expand with diverse data collection modalities as health systems, patients, and community health engagement aim to fill the knowledge gaps toward promoting health and wellness. Here, a conceptual framework is proposed, adapted from the population health framework, socioecological model, and causal modeling in gene–environment interaction studies to integrate the core constructs from each domain with practical considerations needed for multidisciplinary science.

## Graphical abstract

Adapted from the population health framework, socioecological model, and causal modeling in gene–environment interactions, this paper proposes a conceptual framework coined as GxSDoH to represent the interactions between social determinants of health (behavioral, sociocultural, and physical environmental factors) and health outcomes. The framework integrates core constructs from each domain with practical and scale considerations for multidisciplinary COVID‐19 studies.

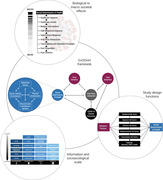

## 1 Introduction

As the COVID‐19 pandemic has ignited public health crises across the globe, attention has been called to how unaddressed vulnerabilities and social needs have exacerbated an already complex problem. Social disparities in health have deeply impacted COVID‐19 morbidity and mortality.^[^ ^1^ ^]^ These disparities are apparent when evaluating available data, which, despite being limited by underreporting of race and ethnicity,^[^ ^1^, ^2^ ^]^ are of utmost importance to understand why the pandemic has disproportionately affected racial and ethnic minorities and other disadvantaged or discriminated against populations in the United States. In particular, high rates of morbidity and mortality have characterized the African American, Hispanic, Native American, and Asian Pacific Islander communities.^[^ ^3^ ^]^

In addition to social factors, there is a need to understand the contribution of genetic factors to COVID‐19‐related health outcomes. Host genetics, such as the ABO blood group and genes related to inflammatory pathways, have been highlighted as potential risk loci for severe COVID‐19.^[^ ^4^, ^5^ ^]^ On the other hand, ongoing research in viral genetics is seeking to understand how strain variations may confer differences in transmissibility, infection severity, and pathogenicity.^[^ ^6^ ^]^ However, host and viral genetics do not occur in a vacuum: they act in concert with socioenvironmental factors that may modify individual genetic risks and phenotypic outcomes. Gene–environment (GxE) interactions help to frame genetic risks in the larger socioenvironmental context in which they occur. When investigating variations in genetic risks across populations, GxE interactions are useful for generating hypotheses and for modeling how disease risk, severity, and outcomes change depending on the interplay of environmental and genomic factors.

With focus on the situation in the United States of America (USA), in this perspective, we aim to provide readers the understanding of the independent and joint impacts of social determinants of health (SDoH) features and genetic risk factors, coined as the GxSDoH encompassing the traditional GxE interactions at the molecular level, on individuals’ vulnerability and susceptibility to COVID‐19 to guide research, health care, and policy related to COVID‐19. However, gaining a better understanding of the interactions between these macro–micro‐level factors is complicated by a series of challenges, which we address in the following sections.

### 1.1 Health Disparities and Social Determinants of Health

Health disparities are defined as the health differences that adversely affect disadvantaged populations based on one or more health outcomes. Health outcomes considered include: 1) higher incidence or prevalence, earlier onset or more aggressive progression of disease; 2) premature or excessive mortality from specific conditions; 3) greater global burden of disease, as measured by population health metrics such as disability‐adjusted life years; 4) poorer health behaviors and clinical outcomes related to the aforementioned; and 5) worse outcomes on validated self‐reported measures that reflect daily functioning or symptoms from specific conditions.^[^ ^7^ ^]^

**Figure 1:**
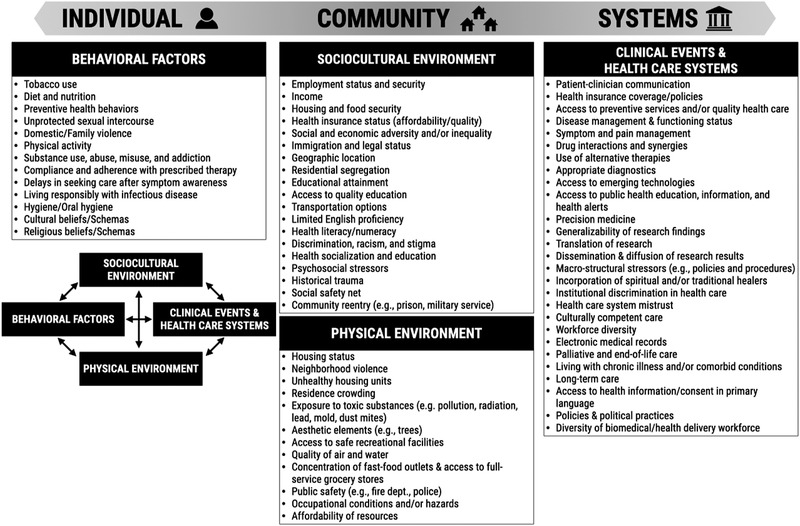
Social determinants of health (SDoH). This nonexclusive list of social determinants of health is categorized into individual‐, community‐, and system‐level factors, with a representation of how these interact with each other.

Health disparities may result from social constructs and behavioral factors, from genetically determined biological features, or from a combination of all. In the context of disadvantaged populations the primary driver of health disparities are the social constructs imposed on these populations. In situations where discrimination has shaped societal organization, social factors significantly impact health outcomes, such that it may lead to stronger inter‐individual variations than those associated with biological and behavioral factors. In these situations, the social factors should not be overlooked when assessing inter‐individual variations. The United States Department of Health and Human Services defines SDoH as “the factors in the environment in which people live, work, learn, and age that affect a wide range of health conditions, functioning, risks, and quality‐of‐life outcomes.”^[^ ^3^ ^]^ **Figure** **1** illustrates how an individual's risks of health outcomes are influenced by an array of behavioral, sociocultural, and physical environmental factors, as well as by clinical events and the health‐care systems the individual has access to^[^ ^3^, ^7^, ^8^, ^9^, ^10^ ^]^ (Figure 1). The list of social determinants of health reported in Figure 1 is nonexhaustive and, for system‐level factors, limited in scope to the health care and biomedical research fields. Moreover, the categorization of many such determinants as individual‐, community‐, or system‐level factors is dependent upon the scale being considered, and thus is hardly univocal.

Several studies have attempted to quantify the impact of SDoH on health. A review by McGinnis et al. estimated that medical care is responsible for only 10–15% of preventable mortality in the US, attributing the rest to other determinants.^[^ ^11^ ^]^ Mackenbach's studies suggest that the true percentage may be even lower.^[^ ^12^, ^13^ ^]^ Both authors stress the importance of assessing the impact of social factors. In a meta‐analysis, Galea et al. suggested that the number of US deaths attributable to a lack of access to quality education, lack of social support, and racial segregation were comparable to the deaths attributable to myocardial infarction, cerebrovascular disease, and lung cancer respectively.^[^ ^14^ ^]^

Among the SDoH, Socio‐Economic Status (SES) is a construct closely related to health disparities. SES includes factors such as income, net worth, and educational attainment.^[^ ^15^ ^]^ Because the US inconsistently monitors SES in terms of health outcomes, it is often challenging to determine the magnitude of its effect. However, research studies of US and European populations have shown that health improves proportionally as social position rises.^[^ ^16^, ^17^, ^18^, ^19^, ^20^ ^]^ Marginalized groups with lower SES characteristics often receive less treatment when compared to their white counterparts, which may contribute further to the disparity.^[^ ^21^ ^]^

While the greatest levels of disparities occur at the intersection of low SES and historically disadvantaged racial and ethnic groups,^[^ ^22^ ^]^ racial and ethnic health disparities persist even in higher SES minority groups^[^ ^15^, ^22^ ^]^ and after controlling for socioeconomic factors.^[^ ^18^, ^20^ ^]^ For example, disparities in birth outcomes between blacks and whites are most pronounced among highly educated women, with highly educated black women experiencing nearly double the rate of infant mortality.^[^ ^23^ ^]^ This implies that health disparities are not attributable to socioeconomic factors alone. In fact, structural racism and discrimination are aspects of SDoH that may directly impact health, wealth, and other social resources, as well as exposure to violence and bodily harm.^[^ ^24^, ^25^ ^]^ A variety of general and disease‐specific mechanisms have been identified linking racism to health outcomes in cardiovascular disease, mental health, birth defects, and other conditions.^[^ ^26^ ^]^ Racism has also been shown to damage health through psycho‐biologic pathways by acting as a pervasive stressor,^[^ ^27^ ^]^ and remains to this day a fundamental cause of persistent health disparities in the United States.^[^ ^28^ ^]^

There are dangers associated with reductive approaches and the naïve inclusion or interpretation of SDoH in analyses. Extra care must be taken, as SDoH often fall along disadvantaged ethnic and racial lines: the effects of ethnic and racial discrimination may be misinterpreted as a mere consequence of genetic correlations. It is therefore essential to distinguish between the concepts of genetic ancestry, cultural ancestry, race, and ethnicity, as their use in biomedical literature can differ from their use in other academic disciplines or from common use (**Table** **1**). In particular, genetic ancestry refers to the description of the population(s) from which an individual's recent biological ancestors originated, as reflected in the DNA inherited from those ancestors.^[^ ^29^ ^]^ Genetic ancestry can be estimated via comparison of participants’ genotypes to global reference populations via the set of genetic variants due to differences in allele frequencies between populations. Cultural ancestry refers to the set of shared cultural characteristics within a group of individuals—characteristics that have little to no underlying causal/biological link to genetic features. or other characteristics. Race and ethnicity are highly context‐dependent, socially driven, and inextricably linked to many social and power dynamics.^[^ ^3^, ^32^, ^33^, ^34^, ^35^, ^36^ ^]^ The term race refers to any one of the groups that human beings are often divided into based on physical traits or ancestry.^[^ ^29^, ^30^ ^]^ Ethnicity is commonly used to refer to large groups of people classed according to common racial, national, tribal, religious, linguistic, or cultural origin or background.^[^ ^29^, ^31^ ^]^ The term genetic ancestry is a modern term specifically used to disambiguate the features (particularly health related ones) arising purely from genetic variation associated with racial and ethnic groups from the social factors that drive the largest health disparities amongst historically discriminated racial and ethnic groups. The terms Race and Ethnicity more often refer to administrative or demographic classifications such as those used by the US Office of Management and Budget for reporting of race and ethnicity for US, federally funded research according to standards set in 1997. This leads to heterogeneous recording of race and ethnicity, because of their context‐dependent nature and differing administrative definitions and uses. Descending from some of such dynamics are racial and ethnic disparities in health care, which refer to the unequal services received or differences in measures of health between populations.^[^ ^21^ ^]^ As we have seen, such health disparities are primarily rooted in nonbiological factors, such as socioeconomic status. However, genetics still plays a role in how diseases manifest themselves and thus can play a supporting role in reducing health disparities.^[^ ^37^ ^]^ With respect to analyzing and interpreting SDoH data, it is incumbent upon researchers to ensure that the relative contributions of both biological and nonbiological factors of disease manifestation are adequately explored.

**Table 1:** Definitions of key concepts discussed in this paper

Demographics	Particular characteristics of individuals or populations. These represent a number of physical, social, economic, and administrative domains. Examples include age, race, gender, ethnicity, religion, income, education, home ownership, sexual orientation, marital status, family size, and disability status.^[^ ^38^ ^]^
Social determinants of health (SDoH)	The conditions in which people are born, grow, work, live, and age, that influence health outcomes, and the forces and systems that shape them. These forces and systems include economic policies and systems, development agendas, social norms, social policies, and political systems. They are shaped by the distribution of money, power, and resources at global, national, and local levels.^[^ ^3^, ^32^, ^34^ ^]^
Race	Any one of the groups that human beings are often divided into based on physical traits or ancestry.^[^ ^30^ ^]^ Race is a culturally and politically charged term, for which definitions and meaning are context‐specific. It is related to individual and/or group identity, and is often linked to stereotypes of visible physical attributes such as skin and hair pigmentation.^[^ ^29^ ^]^
Ethnicity	Large groups of people classed according to common racial, national, tribal, religious, linguistic, or cultural origin or background^.[^ ^31^ ^]^ Ethnicity is used to describe people as belonging to cultural groups, usually on the basis of shared language, traditions, foods, etc. It is often used interchangeably with “race,” and is similarly ambiguous.^[^ ^29^ ^]^
Genetic ancestry	Genetic ancestry refers to the description of the population(s) from which an individual's recent biological ancestors originated, as reflected in the DNA inherited from those ancestors. Genetic ancestry can be estimated via comparison of participants’ genotypes to global reference populations via the set of genetic variants due to differences in allele frequencies between populations. These genetic variants, sometimes called ancestry informative markers, may or may not have biological consequences related to health outcomes, however biological consequences are generally related to variants with Mendelian inheritance patterns that have become prevalent in a population due to founder effects. Different methods of calculating genetic ancestry can yield different results. Genetic ancestry also influences the population distribution of polygenic risk.^[^ ^29^, ^39^ ^]^
Cultural ancestry	Cultural ancestry (or cultural heritage in some definitions) refers to the set of shared cultural characteristics within a group of individuals. These may be religious, political, linguistic, or other cultural traits. Deep cultural ancestry, which is the pattern of shared traits which may persist over hundreds or thousands of years, can be assessed using factors like shared linguistics.^[^ ^40^ ^]^
Racism	Racism is “an ideology of racial domination” in which the presumed superiority of one group is used to accrue power and privilege and justify or prescribe the inferior treatment or social position(s) of others. Racism can be institutional, interpersonal, or internalized.^[^ ^41^ ^]^
Discrimination	Discrimination refers to the unequal treatment of individuals or groups based on some demographic characteristic, such as race, sex, religion, etc.^[^ ^35^ ^]^
Disparity	Disparity refers to unequal outcomes achieved or experienced by different demographic groups (e.g., income, home ownership, education, health, etc.).^[^ ^42^ ^]^

These definitions are representative of the current understanding of these concepts at the time of writing. As the general public and academic understanding of these concepts evolves over time, it is important to frame research questions accordingly.

### 1.2 SDoH and COVID‐19 Outcomes

COVID‐19 acted as a magnifier of pre‐existing health disparities: the pandemic further illuminated how little attention was directed to understand and address the social factors contributing to the disparities.^[^ ^43^ ^]^ The prolonged disruption and lack of a coordinated pandemic response exacerbated the baseline situation, escalating the prevalence of vulnerabilities in all segments of society.^[^ ^44^ ^]^ It is still unclear how the burden of stressors from these events may have shaped individual health, vaccine efficacy, or the very genetics of the affected communities. Some social risk factors, such as essential worker occupations or multigenerational living arrangements, may have conferred risks toward viral infection or may have played a beneficial role toward incident recovery.^[^ ^44^ ^]^ Research into COVID‐19 disparities is challenged with disentangling the SDoH, genetic factors, gene–environmental effects, and their interactions that impacted COVID‐19 outcomes. A few examples of the role played by the SDoH in the pandemic are provided below.

- *Income*: Prior to the pandemic, poverty rates in the United States were 24% for Native Americans, 22% for African Americans, and 19% for Hispanics, compared to 9% for Whites.^[^ ^45^ ^]^ Certain disadvantaged racial and ethnic minority groups, whose financial capacity already constituted a health burden due to high health‐care costs (the median wealth of African American households is ten times less than the median wealth of white households)^[^ ^46^ ^]^ were met with prolonged, deteriorating socioeconomics in the course of the pandemic.^[^ ^47^ ^]^ If sick, some jobs do not provide leave benefits, so individuals face unemployment. As the unemployment rate reached an all‐time high, entire household were at risk of being uninsured due to loss of job‐sponsored health insurance.
- *Working Conditions*: African Americans and Hispanics comprise a disproportionate percentage of essential workers,^[^ ^48^ ^]^ who are not granted flexible work arrangements allowing them to work from home.^[^ ^49^, ^50^ ^]^ In the United States, of the nearly 200k health‐care worker deaths reported by September 21, 2020, a disproportionate number were minorities (case‐fatality ratios: 2.65 for non‐Hispanic Asians, 1.56 for non‐Hispanic Blacks, and 1.14 for Hispanic/Latino).^[^ ^51^ ^]^ These effects are compounded by pressure to go into work despite being sick (contagious presenteeism) in many occupations, due to lack of paid sick leave.^[^ ^52^ ^]^ Indeed, half of the US workforce does not have paid sick leave and disproportionately so in service sector jobs.
- *Living Conditions and Food Insecurity*: Living conditions such as housing insecurity and subpar housing, scarcity of potable water, high crime, and multigenerational households increase the risk of COVID‐19 exposure and/or predispose minorities to worse health outcomes.^[^ ^1^ ^]^ African Americans and Hispanics are also overrepresented among jail populations, which are exposed to crowding and suboptimal living conditions.^[^ ^53^ ^]^ Minorities also tend to have less access to healthy and nutritious foods, which may contribute to negatively affecting their health.^[^ ^1^ ^]^
- *Access to and Quality of Health Care*: Since they are overrepresented in low‐wage jobs that do not provide health benefits,^[^ ^54^, ^55^ ^]^ minorities are less likely than Whites to have access to employer‐provided health insurance.^[^ ^56^ ^]^ Due to structural racism and physician bias, minorities also receive lower quality care: they are, for example, less likely to have their pain appropriately diagnosed and treated and experience longer waiting times to see a provider.^[^ ^57^, ^58^ ^]^ Health‐care facilities that serve areas where disadvantaged groups live also tend to be of lower quality.^[^ ^59^ ^]^
- *Immigration Status, Health Literacy, and Language Proficiency*: Factors often associated with immigration status (such as exclusion from insurance coverage eligibility, mistrust of medical institutions, and limited language proficiency or health literacy) also hinder minorities’ ability to get adequate care.^[^ ^1^ ^]^
- *Environmental Factors*: In the United States, air pollution is among the environmental disparities that are pervasive among racial and ethnic minorities and low SES communities^[^ ^60^ ^]^ by way of occupation, building ventilation or lack thereof,^[^ ^61^ ^]^ permeability of homes,^[^ ^61^, ^62^ ^]^ and closer proximity to pollution sources, such as industrial sites^[^ ^63^ ^]^ or major roadways.^[^ ^64^ ^]^ Air pollution exposure is known to decrease immune function,^[^ ^65^ ^]^ increase local and systemic inflammation due to oxidative stress leading to increased risk of cardiovascular, respiratory, and metabolic disease,^[^ ^66^, ^67^ ^]^ as well as increase expression of proinflammatory genes and decrease expression of anti‐inflammatory genes,^[^ ^67^, ^68^ ^]^ possibly increasing susceptibility to COVID‐19. Other environmental exposures, such as exposure to common water perfluoroalkyl and polyfluoroalkyl contaminants found in up to 98% of the US population^[^ ^69^ ^]^ may also decrease the efficacy of vaccines.^[^ ^70^ ^]^

## 2 Expand GxE to GxSDoH: A Conceptual Framework

**Figure 2:**
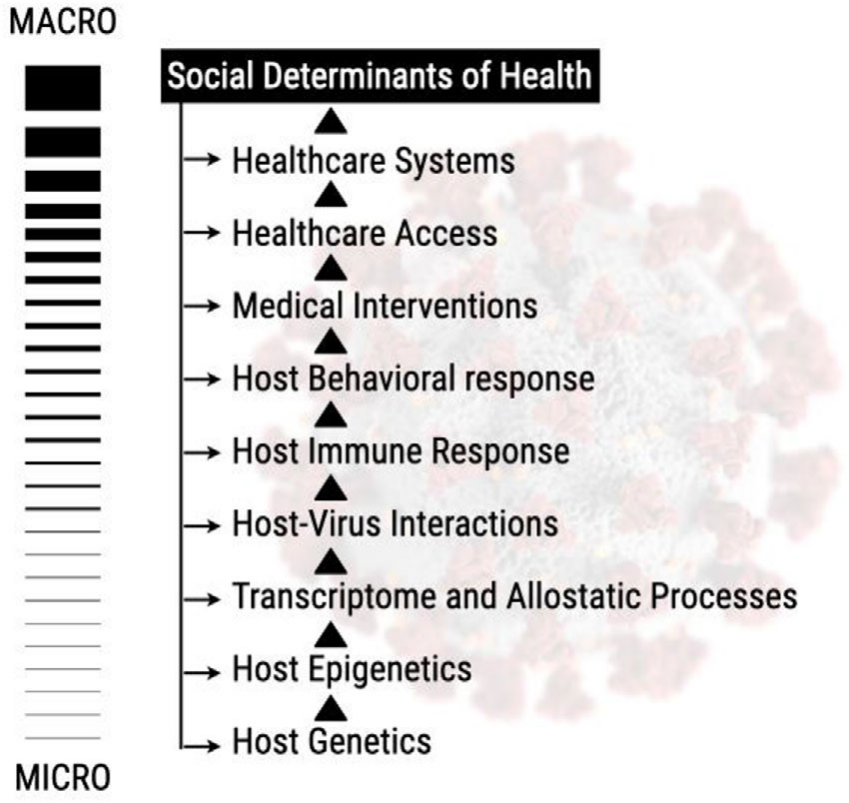
The micro to macro scale of GxE interactions. At the micro level, the host genetics and epigenetics interact to generate the transcriptome and allostatic processes, which manifest at the individual level of host–virus interaction, reflected by the host immune response. Those micromolecular level interactions then interact with or are impacted by the host's behavioral responses, medical interventions, health‐care access and systems, and other SDoH factors.

GxE studies occupy a unique place in epidemiology because they identify individuals and groups whose risk differs from that of either marginal effects, i.e., those with only the genetic feature or the environmental risks alone.^[^ ^71^ ^]^ Key genes perturbed by environmental stressors resulting in disease outcomes have been observed for the effects of smoking,^[^ ^72^ ^]^ air pollution,^[^ ^73^, ^74^, ^75^ ^]^ pesticide exposure,^[^ ^72^ ^]^ lead,^[^ ^72^, ^76^ ^]^ and physical activity.^[^ ^77^ ^]^ In terms of the COVID‐19 pandemic, one needs to consider the GxE interactions at different biological scales, beginning with the host genetics all the way to SDoH that may impact outcomes (**Figure** **2**). GxE studies often require large sample sizes, the availability of cohorts with both genetic and environmental exposures (broadly speaking as even behaviors are included here), and proper study designs. For some exposure effects, a long follow‐up time may be required in order to observe an outcome or identify susceptible time periods of exposure.^[^ ^72^ ^]^ Many observed interactions are either related to absorption or metabolism of a pollutant (e.g., GSTM1 and air pollution for cardiovascular disease)^[^ ^78^ ^]^ or are related to exacerbation of existing risk (e.g., FTO genotypes and physical activity for body mass index).^[^ ^77^ ^]^ Much of the limitations surrounding gene–environment interactions (sample size, longitudinal follow‐up, outcome assessment) could be solved using novel resources like electronic health records (EHRs) or digital health devices. However, most EHR systems do not routinely capture or integrate patient level detailed genetic information beyond targeted testing to explore.^[^ ^79^ ^]^ With increasing availability of genomics data (e.g., biobank data, GWAS, exome and whole genome sequence data) and technologies such as FHIR, more and more clinicians will be able to access or integrate genomic data with EHR data to explore genetic‐phenotypic associations at the point of care.

Recently, adaptations have examined genome‐wide associations and epigenetic changes explained by racial identity as black African American^[^ ^80^ ^]^ or socioeconomic position.^[^ ^81^, ^82^ ^]^ However, these studies may reveal the primary limitation in further SDoH–gene interaction studies, which is that few datasets are available that have collected both relevant genetic data as well as relevant SDoH information at the individual or community level. This may change in the future as the increased usage of area‐level measures for SDoH, e.g., US Census, American Community Survey (ACS), Social Vulnerability Index, allows cohorts initially conceived to study environmental exposures such as air pollution via residential location information, to be adapted to study SDoH. Relevant social factors for understanding the impact of SDoH–gene interactions for COVID‐19 also vary in scale from the individual (essential worker status) to the national (vaccination programs). Within each of these scales there will be a variety of potential biological drivers of the ongoing pandemic that may be revealed through gene–environment interactions and may inform responses to future pandemics. Uncovering these, will require increased collaboration between researchers in SDoH fields and those who generally study GxE interactions. However, hurdles such as mechanistic evaluation in vivo and in vitro models remain.

**Figure 3:**
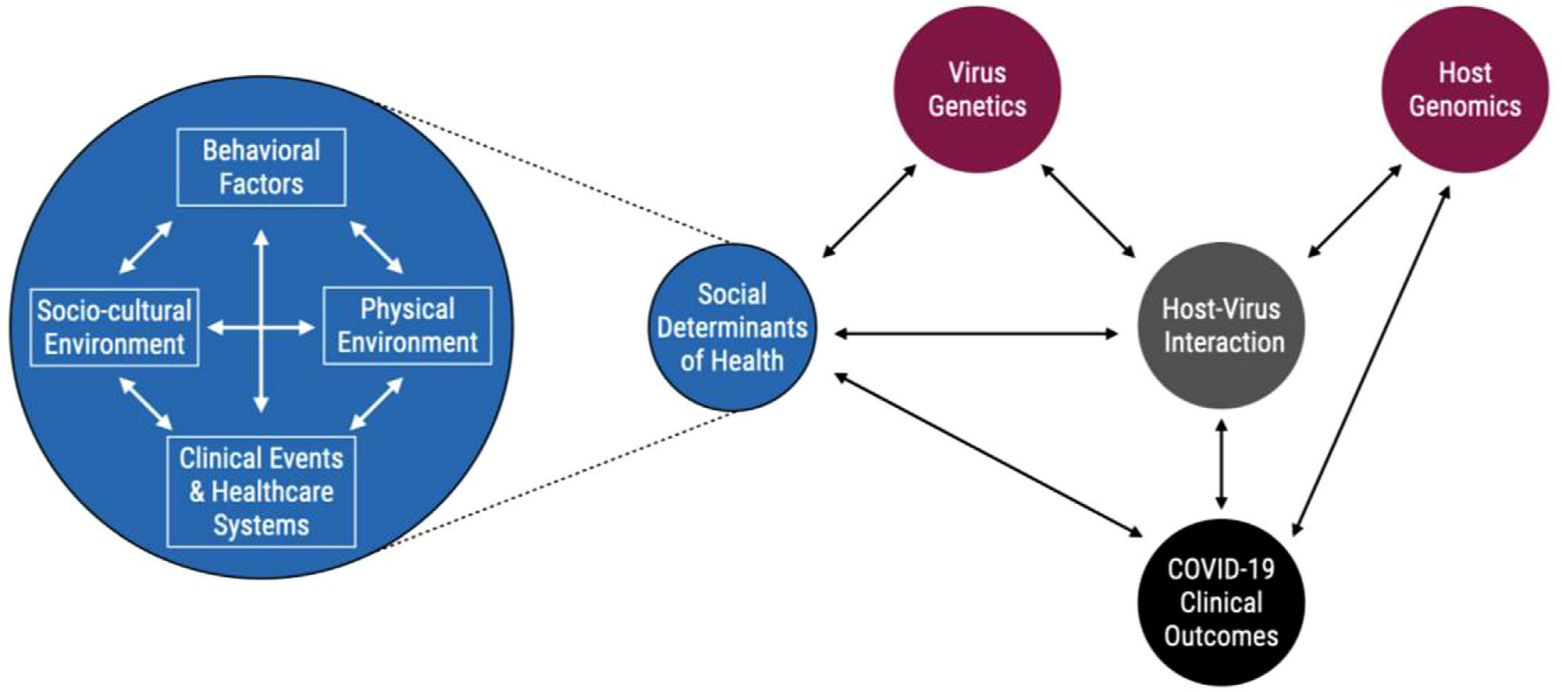
GxSDoH interactions conceptual framework. SDoH encompass personal behavioral, physical‐environmental, social‐cultural, clinical, and health‐care systems factors, which are all interacting with each other. In addition to host genomics, the totality of SDoH or each component of the SDoH interacts with virus genetics, host virus interactions and COVID‐19 clinical outcomes. The virus genetics and host genomics contribute to the host virus interactions. The COVID‐19 clinical outcomes are influenced by the impact of SDoH, host virus interaction and host genomics, and vice versa.

The COVID‐19 pandemic accelerated the global effort of collecting patient level genomics, exposure, and SDoH data. Therefore, the pandemic provided an opportunity to expand the traditional GxE model to the GxSDoH model. However, SDoH features, such as those not necessarily directly mediated through behavior or chemical contaminants, are not frequently studied within the GxE interaction domain. The statistical models that are used to find and describe GxE interactions are perfectly suited to the study of almost any SDoH if the necessary genetic, exposure, and confounder information could be gathered. In fact, using conceptual models for gene–environment interactions such as those developed by Ruth Ottman^[^ ^71^ ^]^ will allow researchers to build GxSDoH models upon the existing conceptual and statistical frameworks for gene–environment interactions. While SDoH do present new considerations in the interpretation of these interactions, the frameworks for considering joint risk from genetic and nongenetic factors will remain largely the same (**Figure** **3**). Figure 3 highlights a potential conceptual framework for GxSDoH interactions that while somewhat expanded, e.g., to include both host and virus genetic/genomic factors, is still consistent with existing GxE models for general gene–environment interactions under the context of COVID‐19. In this framework, SDoH factors impact the dynamics of virus genetics, host–virus interaction, and COVID‐19 clinical outcomes, and vice versa. The increased susceptibility of the host due to genomic factors such as epigenetics, which are in part driven by SDoH, impacts the host–virus interactions and the COVID‐19 outcomes that interact with and are influenced by SDoH. The COVID‐19 clinical outcomes are determined by the interplay of SDoH, host–virus interaction and host genomics. Many mathematics or statistical models can be drawn from this conceptual framework to study COVID‐19 and SDoH.

## 3 Genetics of COVID‐19

SARS‐CoV‐2 and COVID‐19 are still novel, and there are significant gaps in our knowledge about virus genetics, how virus‐host interaction may be influenced by host genetics, and how GxE might impact either or both. Here we highlight some of what is known on the topic, and how SDoH might play a role in our understanding and be incorporated into future research.

### 3.1 Host–Virus Interaction and Social Determinants

The SARS‐CoV‐2 mRNA virus, with a genome of ≈30k nucleotides, encodes for structural proteins, such as the spike (S), envelope (E), membrane (M), and nucleocapsid (N) proteins.^[^ ^83^ ^]^ The virus’ genetic diversity, pilfered from host cellular ingredients, determines its transmissibility, pathogenesis, and virulence. As the SARS‐CoV‐2 virus proceeded to evolve, multiple new strains potentiate concerns of transmissibility and pathogenicity.^[^ ^84^ ^]^ In South Africa, the 501Y.V2 strain (later known as the SARS‐CoV‐2 “beta variant”) emerged in October 2020 as substantially more transmissible than the original observed strain. Although hospitalization rates appeared to be similar between the 501Y.V2 strain and previous strains, in‐hospital mortality escalated 20% higher with the 501Y.V2 strain.^[^ ^4^ ^]^ Also detected around October 2020, the B.1.617 strain, also known as the SARS‐CoV‐2 “Delta variant,” has mutated spike proteins similar to the Alpha and Beta variants but has shown to be more virulent and pathogenic to the infected hosts. Since then, Delta has become prolific, adopting genetic variations between infected hosts, meriting new concepts of classifying the viral lineage as Delta‐plus. On 26 November 2021, WHO designated the variant B.1.1.529 a variant of concern, named Omicron. The S protein of the Omicron variant harbors an unusually high number of mutations, which may contribute to Omicron's higher ability of infecting convalescent individuals as compared with previously circulating variants. Hoffmann et al. also reported the possibility of the Omicron variant's ability to evade the neutralization by antibodies from vaccinated individuals.^[^ ^85^ ^]^

COVID‐19 gives us an example of how pathogen genetics may interact with SDoH to alter related health outcomes. Often, marginalized communities such as foreign‐born workers are more likely to be classified as “essential” workers compared to the native‐born population in the USA.^[^ ^86^ ^]^ Essential workers are far more likely to test positive for SARS‐CoV‐2 than the general community,^[^ ^87^ ^]^ which means they are more likely to be exposed to, contract and, consequently, be hospitalized with new, more transmissible, and more deadly viral variants.^[^ ^86^ ^]^ Thus, understanding COVID‐19 morbidity and mortality risks in these individuals is dependent both on their likelihood of being exposed and the genetic makeup of the viral variant exposure which may differ from that in the general population. This public health risk, which may be best modeled as an SDoH–pathogen–genetics interaction, is key to effective deployment of resources to communities more likely to be infected with more pathogenic versions of the virus. It is important to note that this SDoH–pathogen–genetics interaction may also extend to the household in which essential workers from marginalized groups live as household members. The emergence, prevalence, and various transmission rates of SARS‐CoV‐2 variants among different countries or continents shows another evidence of social, political, and public health measures impacting the virus’ genetic evolution.

### 3.2 COVID‐19 Outcomes and Genetic Susceptibility

Host genetic variability plays a role in determining an individual's susceptibility to COVID‐19 and likelihood to matriculate worse health outcomes. Substantive research has explored the role of key genetic markers in the infection of Angiotensin‐Converting Enzyme 2 (ACE2)^[^ ^88^, ^89^ ^]^ and Transmembrane serine protease 2 (TMPRSS2),^[^ ^89^, ^90^ ^]^ though these sources of diversity have not exposed notable polymorphic attributes to protect the susceptible. For example, genetic variability in the immune system across the 3 major histocompatibility complex (MHC) class I genes have been shown to affect an individual's susceptibility to and severity of COVID‐19.^[^ ^91^ ^]^ The potential role of epigenetic modulation along the renin‐apoptosis and pro‐inflammatory pathways activated by SARS‐CoV‐2^[^ ^92^, ^93^, ^94^ ^]^ has also been hypothesized to explain worse health outcomes in patients with pre‐existing comorbidities, such as Diabetes.^[^ ^95^, ^96^ ^]^ Diabetes elevates activation of the renin‐apoptosis and pro‐inflammatory pathways, which are targeted by SARS‐CoV‐2,^[^ ^92^ ^]^ highlighting the hypothetical role of epigenetic modulation and why some patients may be predisposed to develop more severe COVID‐19 or experience the sequelae at higher rates once infected. Once exposed, SARS‐CoV‐2 activates its receptor and modulators, the ACE2 and TMPRSS2, eventually triggering the immune response, potentially multisystem inflammation, apoptosis, and further transmission to other hosts.^[^ ^88^, ^90^ ^]^

### 3.3 Pandemics and Other Mass Casualties Exacerbated by GxE Interactions

Epigenetic GxE interactions may have unique influence morbidity and mortality of COVID‐19 patients, as “prepandemic” epigenetic states may result in health‐relevant GxE interactions as well as epigenetic states altered by the stress of the pandemic, which may exert effects on this generation and beyond. Apart from the immediate health effects, traumatic loss and widespread disruption from the pandemic are comparable to historic tragedies and disasters, where environmental exposures have been associated with multigenerational health risks transmitted epigenetically along stress pathways.^[^ ^97^, ^98^ ^]^ For example, chronic exposure to racism has been associated with poorer health outcomes among African Americans and their offspring due to elevated cortisol during pregnancy, increasing risk of preterm births and low birth weight, leading to a higher risk of metabolic dysregulation as the infants become adults.^[^ ^99^ ^]^ Similarly, exposure to environmental pollutants can result in epigenetic changes, as seen Hispanic farm workers exposed to pesticides, which lead to increased risk of metabolic disorders trans‐generationally,^[^ ^100^ ^]^ implying a potential epigenetic mechanism. During the Dutch famine of 1944–1945, prolonged nutritional deficits among women who were pregnant during this period is associated with multiple generations of offspring with increased neonatal adiposity, increased adult BMI, and overall poorer health.^[^ ^97^ ^]^ Thus, in a GxE model historical (possibly even in previous generations) socially driven stressors may be both inducers of particular epigenetic states—some of which are certain to confer higher risks—which may interact with current experienced environmental social factors, such as lack of available hospital resources in lower‐income areas, to alter COVID19 outcomes. Thus, we would have historical social stressors manifesting as a genomic factor, the “G,” and combining with current social environment (“E”) to jointly impact COVID19 outcomes. However, care must be taken in assessing transgenerational effects as biases with health‐care access may confound the association. Furthermore, the breadth of longitudinal effects from the COVID‐19 pandemic are yet to be studied.

## 4 Practical Considerations for GxSDoH Research

### 4.1 Leveraging Traditional GxE Tools and Methods for GxSDoH

**Figure 4:**
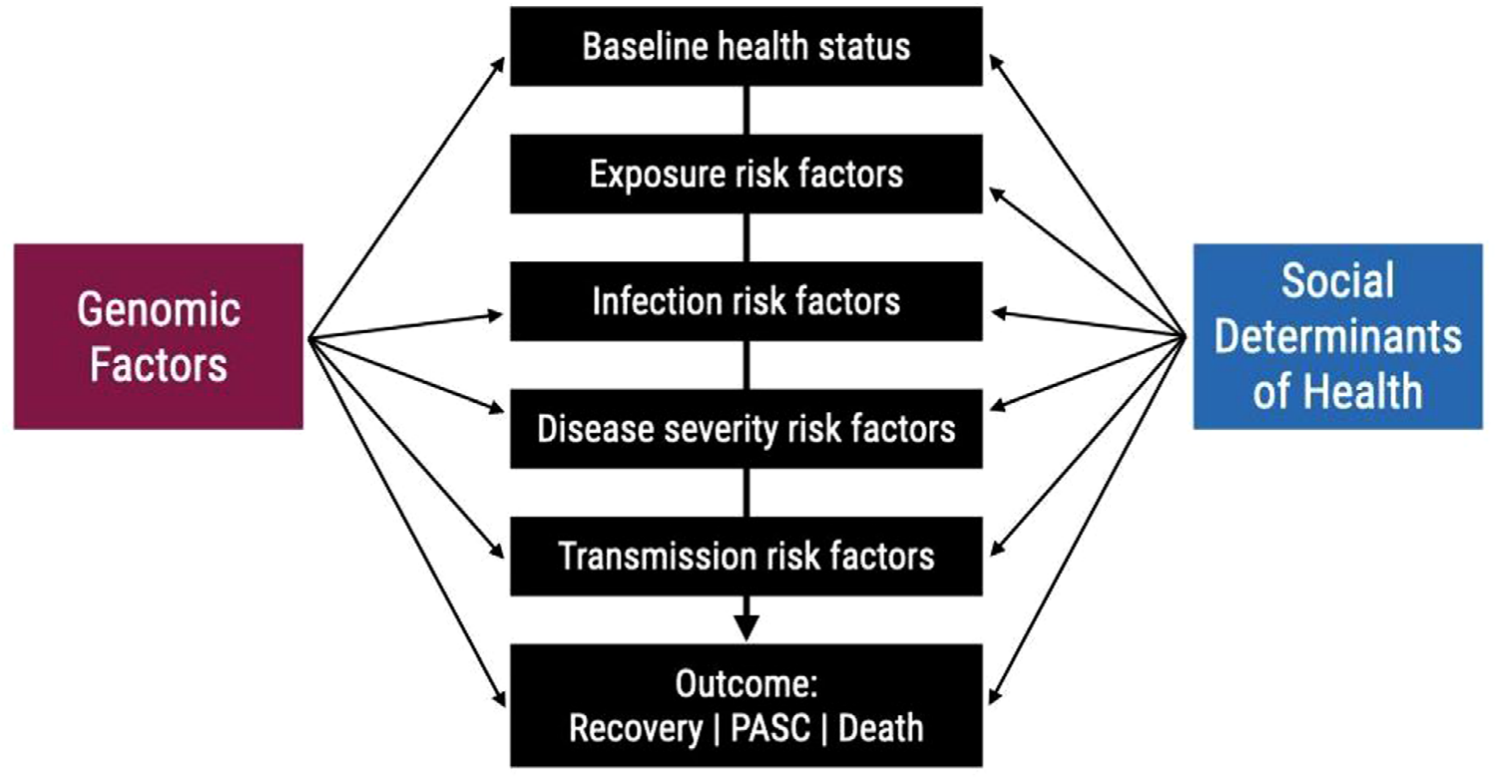
Scale of multicollinearity between social determinants of health and genomics (100–102). The intersection between genomics factors and SDoH can occur at multiple points in the disease process and influence the exposure and outcomes of interest.

Research focusing on GxE interactions has integrated many tools and methods borrowed from a variety of fields, from epidemiology to molecular genetics. These tools and methods require that both the susceptible genetic make‐up and the environmental exposures are present to garner a response. SDoHs include a wide variety of social influences that impact outcomes differently. This complicates their study. However, there are several clinical and research targets that may intersect both GxE and SDoH research (**Figure** **4**).

Some examples of tools used in GxE analysis, that could be used as a template for SDoH research, include CGEN, the PhenX Toolkit, and the National Center for Biotechnology Information's (NCBI) database of Genotypes and Phenotypes (dbGaP). CGEN is an R package for GxE analysis and is an open‐source codebase useful for reproducibility in assessing genome‐wide and cumulative risk for SNPs in case‐control data.^[^ ^101^ ^]^ The PhenX toolkit is a repository of protocols to assist in standardizing human genomic research,^[^ ^102^ ^]^ and establish methods to assess the GxE interactions, including SDoH and COVID‐19.^[^ ^103^ ^]^ The PhenX toolkit is searchable and is compatible with dbGaP so researchers can easily utilize the protocols to suit their needs. These protocols, which can play an important role in research design as in vitro/in vivo methods, are hard to adapt for SDoH. The NCBI dbGaP is a registry system for meta‐data about the genotypic and phenotypic studies for controlled access data. It is a very rich resource for the phenotypic and measurement data for genomic studies. However, the dbGaP contains very limited SDoH data elements, such as smoking status, education level and maybe income level. There are criticisms about the subpopulation and conditions represented by the dbGaP data.^[^ ^104^ ^]^ To date, there is currently no database collecting SDoH data and genomics data as comprehensive as dbGaP collecting phenotypes and genotypes data. Newly created COVID‐19 genomics databases, such as the COVID‐19 Host Genetics Consortium, may be a venue to start collecting SDoH and genomics data systematically for SDoH and COVID‐19 research.

Due to the inherent interactions between SDoH, a variety of statistical and modeling methods will be required to parse out the meaningful role and directionality contributed from each interaction.^[^ ^71^, ^79^ ^]^ Besides the aforementioned tools, definition of the interaction scale is a critical consideration that guides the methodology and GxE analysis plan. Within GxE interactions, additive and multiplicative interactions have larger magnitudes of exposure effect that differs between genotypes.^[^ ^71^ ^]^ Multiplicative and synergistic interaction provides more useful information in discerning the impact of an exposure on disease whereas additive interactions are more useful in identifying disease prevalence. Existing GxE and epidemiological models provide frameworks to evaluate additive and multiplicative interactions; additional modeling methods are needed to account for biases associated with sampling approach, individual versus community‐level findings, and the complex variability not explainable by singular or paired interactions alone.

### 4.2 GxSDoH Informatics: Data Collection, Data Sources, Quality, Methods, and Standards

GxE studies require large sample sizes to detect an effect and can be computationally expensive. Additionally, there are multiple study designs and analytic models to consider in GxE analyses.^[^ ^96^ ^]^ Chemical environmental exposures are often complex and multifaceted, requiring knowledge about the chemical(s) and their bioavailability, dose, route of exposure, metabolism/excretion, and timing of exposure. SDoH can be more complex, requiring knowledge about the society in which they occur, individual‐level and area‐level indicators, and timing of SDoH exposure with respect to the outcome being observed. In addition, the social system's structural level factors such as public health policy and health systems add to the complexity of SDoH research. Researchers often lack the tools and resources to collect the fine granular level of the SDoH data over the course of an individual's lifetime, and instead often use aggregate data from publicly available sources.

While genetic and genomic data are on track to make data FAIR (findable, accessible, interoperable, and reusable,^[^ ^105^ ^]^ SDoH data is far from this reality. Basic demographic information provides a glimpse of a patient's social determinants (e.g., occupation, postal code, education level). A challenge unique to SDoH is that these data can be collected at many levels (e.g., patient, neighborhood, community, county) and across subject areas (e.g., education, economic stability, housing instability, and discrimination). Historically, biological samples, clinical findings, epidemiological observations, environmental exposure, and social‐economic knowledge have existed in separate silos, if at all, yet they are fragment representations of the same subject's reality. With broader adoption and advancement of EHRs, the linkages and data representation of biological samples and clinical findings now yield near‐real‐time public health use cases, such as surveillance of COVID‐19 cases. Consortia such as the National COVID Cohort Collaborative (N3C) have expedited the creation of this kind of research‐ready data sets. The movement toward understanding more macroscale social and environmental factors with policy implications and more microscale epigenetic phenomena for medical innovations continue to be functional works‐in‐progress (Figure 2). Due to their documented importance in areas of health, they remain a potential source of unmeasured confounders in clinical and genetic COVID‐19 research. Moreover, the quality of SDoH data is often poor and affected by the differential reporting of the effects of the pandemic upon racial and ethnic communities.^[^ ^107^, ^108^ ^]^ Unfortunately, the collection of individual‐level SDoH is often missing from COVID‐19 research, with missingness especially affecting race and ethnicity data (47% and 43% of cases, respectively).^[^ ^106^, ^107^ ^]^ Overall, we would like to emphasize that while we hope the FAIR principles are adopted by more data repositories, it is not clear if there exist many datasets that tightly align with FAIR ideas. However, there are some efforts that come close to the high‐level guidelines of FAIR. The N3C COVID patient dataset is one such resource with elaborate harmonization of clinical data from over 65 facilities (across the US) into a common data model along with indexing/retrieving capabilities and reusable pipelines.^[^ ^108^ ^]^ This FAIR N3C COVID data is accessible via a secured cloud enclave environment.

**Figure 5:**
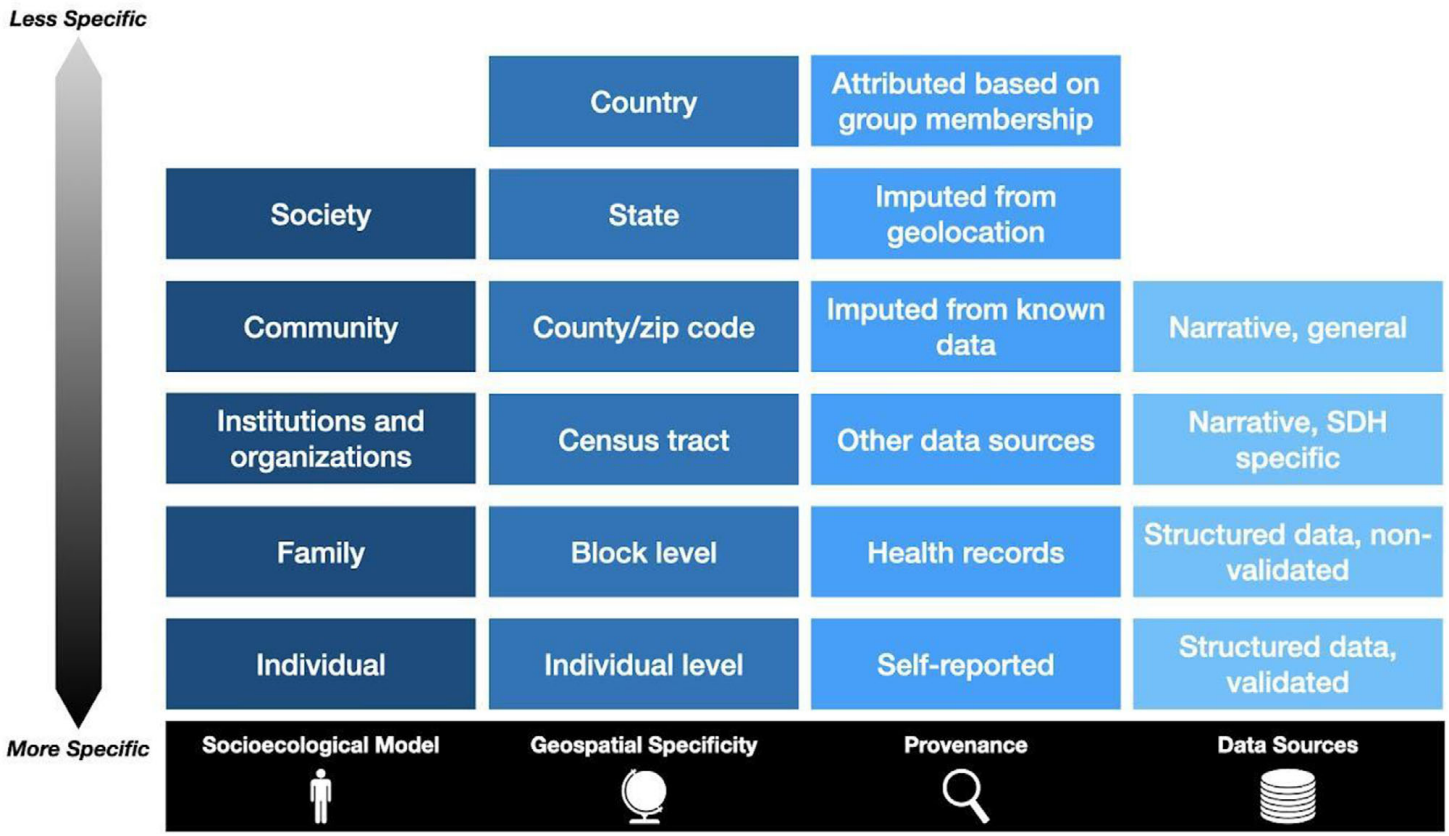
Dimensions of social determinants of health data to consider when selecting data sources and analytic approach, ranging from more to less specific or granular.

The choice of SDoH data sources and analytic approach can be complicated by several aspects of the data's granularity and provenance. Researchers may have more controls of some of these variables when there is primary data collection as opposed to secondary use of clinical data. Some of the critical factors (**Figure** **5**) to consider include:

- *Socioecological Model*: Individuals exist nested within increasingly complex and dynamic systems and communities. Researchers who are exploring the influence/role of SDoH should be aware at what level and through which mechanisms a given determinant of health interacts with or impacts an individual.
- *Geospatial Specificity*: SDoH data can be collected directly from individuals, but it can also be imputed using geospatial systems and contextual data from sources like the ACS. In the USA, depending on the variable of interest and the data source, these can be available from census blocks (600–3000 individuals), census tracts (1200–8000 people), zip code or county, state, and national‐level. In other countries, there are similar geospatial divisions. Researchers should carefully consider how to interpret and use data that was obtained using geospatial methods, as it is often true at the aggregate level, but not necessarily at the individual level.^[^ ^109^, ^110^ ^]^
- *Provenance*: How SDoH data were obtained can give insights into how reliable it is. Data that are self‐reported by the patient are often considered to be the most reliable. Information recorded or reported by a health‐care provider in the record is affected by being filtered through at least one person, and subject to those individual biases. Other data sources may be even less reliable than medical records, which at least are subject to medico‐legal requirements of accuracy and veracity. Imputing data from known facts or geolocation provides an exciting opportunity for novel research approaches but should be considered in context. Attributing a factor based on membership in a group (e.g., shorter projected lifespan for an African American individual) is the least reliable method and not recommended.
- *Data Source*: Structured, validated instruments like PRAPARE and Health Leads are specifically designed to capture data about SDoH. EHRs often capture other elements of SDoH in other structure fields such as social histories and patient questionnaires, though these may not have been designed or validated for SDoH data collection specifically. Free text narratives such as patient histories or notes from social workers and mental health professionals may include a significant amount of information relating to SDoH but requires interpretation and coding, which can introduce errors. Finally, SDoH may be captured in other free text elements of the medical record, but its value may be offset by the amount of effort required to search through entire records.
- *Temporal Analysis of Exposure to SDoH* (*not pictured in* *Figure* *5*): Major questions remain about the best way to incorporate time as a variable with social determinants of health data. Few standardized SDoH instruments include questions about length or time of exposure. Many public data resources like the ACS, EPA, and USDA include the year of collection, but complicated rolling average data releases can make it difficult for researchers to understand which estimates to use for different “exposure windows” for individual patients. The literature on Adverse Childhood Experiences (ACEs) further highlights the importance of when in an individual's life they had specific exposures.^[^ ^111^ ^]^ Researchers will need to work collaboratively across disciplines to identify spatiotemporal analysis models that are fit‐for‐use for SDoH.^[^ ^112^ ^]^

#### 4.2.1 Health‐Care‐Related Sources of SDoH Data

*Structured Data Collected under Normal Hospital Operations*: These include data collected as part of patient registration and care delivery, including demographics, insurance type and status, and preferred language.^[^ ^113^, ^114^ ^]^ Some clinical notes may contain structured fields related to SDoH, such as employment and housing; however, these structured fields may not be designed to be easily searchable or extractable. Moreover, standard diagnostic codes for SDoH and social needs findings exist in ICD‐10, but these codes have rarely been used.^[^ ^114^ ^]^ In the USA, the Meaningful Use legislation “American Recovery and Reinvestment Act of 2009” requires the following demographic fields: preferred language, sex, race, ethnicity, date of birth^[^ ^115^ ^]^ to be captured in an EHR system. The HL7‐FHIR Gravity Projects have focused on concept representation efforts such that this information can be relayed for adoption in Public Health workflows.^[^ ^116^, ^117^ ^]^ Since it is kick‐off in May 2019, the Gravity Projects have proposed standardized SDoH data elements to USDI v2.0 and SDoH terms to ICD‐10 covering the critical SDoH domains described by *Food Insecurity*, *Housing Instability and Homelessness*, *Inadequate Housing*, *Transportation Insecurity*, *Financial Strain*, *Social Isolation*, *Stress*, *Interpersonal Violence*, *Education*, *Employment*, and *Veteran Status*. This work is rapidly evolving while the COVID‐19 pandemic prolongs. It is worthy to follow the implementation and evaluation of these codes in EHR systems.

*Unstructured Data in Clinical Notes*: Free text documentation by clinicians often contains a wealth of information characterizing a patient's living experience that directly addresses the SDoH in their lives. Unfortunately, to extract high quality data from those clinical notes requires the advanced natural language processing (NLP) techniques and manual chart review, both of which are labor‐intensive, expensive and do not scale well.^[^ ^113^ ^]^ Recent developments, such as Machine Learning (ML) and Artificial Intelligence (AI) use in NLP may alleviate the scalability issues, but their accuracy varies depending on the particular SDoH attributes; hence NLP methods need extra evaluation and validation before real world use.

*Patient‐Level SDoH Screening Tools*: a number of institutions have started screening patients for SDoH and social needs using standardized survey instruments, such as PRAPARE, WeCare, Health Leads, and others.^[^ ^118^, ^119^, ^120^ ^]^ This may be done systematically for all patients to capture SDoH data at a granular level that impacts a patient. The major barriers for extracting this data are how the instruments were built into the EHR, and how uniform the data collection is across the patient population, and the controlled vocabulary to compare across screening tools. Although many of these instruments have been validated, few of them have been mapped to existing ontologies or controlled vocabularies. Namely, the standardization of those surveys remains challenging. In addition, while time (when an exposure happened and for how long) is an important variable to consider, none of the above data sources address it in a meaningful way.^[^ ^121^ ^]^ Researchers will likely need to develop standardized SDoH data collection instruments, like questionnaires, that are built‐for‐purpose to capture all relevant dimensions of GxSDoH interactions.

*Contextual Data Using Geocoding*: A patient's home address can be used to link the patient to a geographic boundary of varying granularity (neighborhood, census tract, zip code, county, and state), which can then be used to identify SDoH information from existing databases, such as the American Community Survey (ACS).^[^ ^122^ ^]^ While this approach is particularly useful in population health efforts, geocoded contextual data should be used and interpreted carefully at the individual patient level.^[^ ^123^ ^]^

*Mobile Health and SDoH Data*: The proliferation and adoption of digital health tools, including mobile health apps and wearable sensors, holds great promise for improving human health.^[^ ^124^ ^]^ As of 2019, there are between 400 000 to 500 000 health, wellness and fitness apps that run on smartphones, watches, tablets, and other mobile devices, available for download from platform‐specific application stores such as the Apple App Store (iOS) and Google Play (Android).^[^ ^125^ ^]^ Prospective SDoH research could incorporate these digital health innovations for collecting geospatial‐temporal data to understand individual‐level physical activity and behavior, exposure duration and potentially dosage, and population‐scale influences from environmental factors. For example, while aiming to establish a long‐term patient cohort to study the postacute sequelae of SARS‐CoV‐2 (PASC), the NIH RECOVER program (https://recovercovid.org) supports a scalable, configurable, and integrated Mobile Health Platform to provide RECOVER studies with customized mobile apps and to enable the secure collection of PASC digital health measures.

*Other Patient Cohort Data*: A silver lining of the current crisis is an increased awareness of the importance of discovering, collecting, and standardizing SDoH data. Many working groups have been formed to focus on making SDoH data ready for research or decision making.^[^ ^116^, ^126^ ^]^ Some national and international‐wide longitudinal cohort consortia provide patient‐level genomics data and clinical data. This kind of data is promising for SDoH health research when SDoH data can be integrated with the cohort data. For example, the (United Kingdom) UK Biobank is a very large, population‐based prospective study with over 500 000 participants aged 40–69 years (at the time of recruitment in 2006–2010). The UK Biobank is established to combine extensive and precise assessment of exposures with comprehensive follow‐up and characterization of many different health‐related outcomes.^[^ ^127^ ^]^ The Townsend deprivation index at recruitment is calculated at the zip code area for each participant. The All of Us Research Program is a longitudinal cohort initiative aimed at advancing precision medicine and improving human health by enrolling at least 1 million diverse individuals across the United States. The program has prioritized the recruitment of participants from populations that have been historically underrepresented in biomedical research. The All of US program launched the COVID‐19 Participant Experience (COPE) survey May 7, 2020 to incorporate SDoH data.^[^ ^128^ ^]^ The Million Veteran Program is another observational cohort study and mega‐biobank, with 775 000 Veteran partners as of August 2019, in the Department of Veterans Affairs health‐care system. In addition, the International HundredK+ Cohorts Consortium (IHCC) is a collaboration of 61 large scale cohorts located in 32 countries, with total current sample sizes across all cohorts of roughly 30 million.^[^ ^129^ ^]^ Although an increase in awareness of the importance of SDoH has led to more organizations collecting this type of data, it still tends to be sparse in most health information systems.^[^ ^106^ ^]^

#### 4.2.2 The Reality of Doing (GxE) Studies during a Global Pandemic

In order to quickly understand and tackle COVID‐19, which continues to have high morbidity and mortality, the normal routes of conducting research have been expedited so scientists could share their results and build upon the growing foundation. This has led to many cross‐continental collaborations across disciplines and innovative means of conducting research. However, it comes with logistical and ethical concerns. For example, privacy concerns^[^ ^130^ ^]^ or the risk of inadvertently uncovering protected health information (PHI) through geocoded features, increasing data dimensionality, or the use of non‐EHR data, such as social media. Informed consent and information sharing practices have been accelerated, and preprint publications are available long before peer reviewed journals have accepted them, often leading to confusion when poor quality or erroneous work is picked up by mainstream media.^[^ ^131^ ^]^

Of concern for SDoH data is that the quality of that data is often poor, and may be missing not at random^[^ ^106^ ^]^ as with missing or misidentified race and ethnicity data, which are greater for black or Hispanic patients within EHRs.^[^ ^107^ ^]^

## 5 Limitations and Generalizability

The SDoH information presented here is applicable to research within the USA. However, observations of negative health outcomes and disparities affecting historically disadvantaged groups as well as increased environmental exposures for lower Socio‐Economic Status are a global phenomenon. GxE interactions often assess genetic variants, which can have higher frequencies in specific ethnicities.^[^ ^132^ ^]^ Environmental exposures can occur at chronic or intermittent low doses in some populations because some Socio‐Economic Status or race and ethnicity groups may have a higher likelihood of exposure. Not only can both factors require large study populations and lengthy follow‐up times in order to note an effect, but they can also introduce bias and therefore limit the generalizability of the results to populations with similar genetic variant frequencies and exposure distributions.

Limitations related to data used in GxE interactions research and SDoH data include the lack of diversity within genomic data sets.^[^ ^104^ ^]^ Within two large genomic studies, the dbGaP and the Genome‐Wide Association Study (GWAS) catalog, less data was available for those who are not of European or Asian ancestry.^[^ ^104^, ^133^ ^]^ Missing racial and ethnic data is an issue seen when COVID‐19 data are reported to the CDC as this differential reporting limits our knowledge about incidence and vaccine coverage within racial and ethnic communities.^[^ ^2^, ^107^ ^]^ The data processes generating SDoH measures may not always be transparent, which could lead to data quality issues.^[^ ^134^ ^]^ The periodicity of data collection is not consistent across measures and some measures will be more up to date than others. Additionally, definitions of SDoH domains vary, whereby comparisons can be challenging due to differences in the information collected.

There is a long and ongoing history of failure to meet the Belmont principles of respect for persons, beneficence, and justice for nonwhite individuals in the United States. These failures have included, but are certainly not limited to, lack of informed consent, unethical experimentation, and underrepresentation. These failures exist in a society that continues to perpetuate systemic racism and prejudice that relies on and results in significant disparities in power and agency. American Indians and Alaska Natives are under‐reported in COVID‐19 public health surveillance data, if at all.^[^ ^135^ ^]^ The importance of data sovereignty, ownership, and decolonization has been highlighted especially by and regarding American Indians and Alaska Natives.^[^ ^136^ ^]^ Unfortunately, it is common for researchers to exclude members of underrepresented communities rather than address the relevant concerns, leading to further inequity. This is especially problematic during the COVID‐19 pandemic. The Urban Indian Health Institute states that “current standard data collection practices by many federal, state, and local entities effectively omit or misclassify American Indian and Alaska Native populations, both urban and rural. This is particularly concerning in the midst of the COVID‐19 pandemic as these current standards of practice are resulting in a gross undercount of the impact COVID‐19 has on Native people”.^[^ ^137^ ^]^ Practical approaches for these evolving and complex issues exist at the intersection of legislation, ethics, and biomedical research. Therefore, research focusing on genetics and health outcomes of members of historically disadvantaged racial and ethnic groups must be informed by—and ideally conducted in collaboration with or under the leadership of—representatives of those groups.

It is promising to see that the NIH is taking actions to specifically support data sharing, data management planning, and research governance for equitable use of tribal data and minority communities. For example, the NIH's notice on the Final NIH Policy for Data Management and Sharing mentioned that NIH is “developing supplemental information for researchers who wish to work with AI/AN communities”.^[^ ^138^ ^]^ The NIH also initiated the AIM‐AHEAD program,^[^ ^139^ ^]^ which aims to increase the participation and representation of researchers and communities currently underrepresented in the development of AI/ML models and enhance the capabilities of this emerging technology, beginning with EHR data and via meaningful, mutually beneficial partnerships.

Another caveat to consider is the risk of data leakage and associated privacy concerns especially to individuals from vulnerable segments of the society. Although there are benefits to including datasets from minority populations in terms of having better analyses and models that may eventually help these communities, the risk of disclosure of sensitive personal health information may be too high. Apart from several state laws, the United States does not have a singular federal law that covers the privacy of all types of data. In contrast, the European privacy law, General Data Protection Regulation (GDPR),^[^ ^140^ ^]^ requires companies to ask for some permissions to share data and gives individuals rights to access, delete, or control the use of that data. The ethical, legal, and societal implications of genomic data sharing is another area that is also rapidly evolving. As the landscape of data protection guidance and laws evolve, the challenge is to ensure that outcomes of such research or proposed policies remain compliant with Belmont principles, prevent harm and promote benefit to the communities and enhance their vulnerabilities. Meanwhile, we have several strategies to mitigate risk of disclosure and associated harm: 1) the process of de‐identification, by which identifiers are removed from the health information, mitigates privacy risks to individuals and thereby supports the secondary use of data for comparative effectiveness studies, policy assessment, life sciences research, and other endeavors.^[^ ^141^ ^]^ 2) An additional strategy to handle inadvertent disclosure due to small counts is typically handled by data governance and stewardship committees that require that patient counts larger than a particular minimum value (say, 20) be reported in presentations/manuscripts. 3) Risk due to adversarial players in the context of machine learning and AI methods can be mitigated through a combination of federated learning (data visiting), where raw data is never really shared, and learning with differential privacy guarantees, where data leakage is statistically minimized to a user specified threshold.^[^ ^142^, ^143^ ^]^

Lastly, the existing gap in health outcomes should not widen into greater disparity, especially in the age of precision medicine. This motivates an inclusive approach to genomic, clinical, and medical research, enabling participation from all racial, ethnic, and economic strata for the benefits of precision medicine to be fully realized.

## 6 Conclusion

SDoH explains how certain needs and risk factors disproportionately burden the ability to promote well‐being and prevent worsening health outcomes in segments of the community‐at‐large.^[^ ^3^, ^144^ ^]^ In the United States, enduring health disparities stem from unaddressed social needs, creating clusters of under‐resourced communities and neighborhoods vulnerable to catastrophe and at greater risk of mortality and burden of disease.^[^ ^3^ ^]^ In essence, the historical lack of investment to address social needs has created baseline conditions for the virus to spread quickly where social distancing was challenging or impossible. Areas with high rates of baseline comorbidities were vulnerable to more severe COVID‐19 outcomes.^[^ ^145^, ^146^, ^147^ ^]^

In the context of COVID‐19 and future pandemics, it is key to understand the complex interplay between the genetics and epigenetic variations with SDoH and how intersectional research data can be designed for reproducible and generalizable research. First, all biomedical researchers must understand the difference between demographic concepts, like race and ethnicity, biological factors like genetic ancestry, and the societal experiences with which they are associated. Biological and genetic factors can elicit behavioral responses observable at the individual level. As such, behavioral factors respond to but also influence the physical environmental and sociocultural environmental factors. Clinical events and health‐care systems provide a safety net and supportive role to address social needs, though resource limitations and barriers to access prevent meaningful intervention and health promotion.

GxE interactions are a central means by which to understand how genetic, and more broadly genomic, states of the individual may be influenced by responses to environmental exposures in addition to responding to them.^[^ ^148^ ^]^ Liu et al. found that heritability estimates can be biased when environmental factors are not accounted for.^[^ ^149^ ^]^ Similarly, identifying how much population genetics contributes to health must not neglect the SDoH factors.^[^ ^150^ ^]^ Attempts to incorporate SDoH should not be taken lightly as there is significant debate regarding the impact of incorporating genomic research in efforts to reduce health disparities.^[^ ^151^ ^]^ Though genomic researchers are beginning to incorporate SDoH factors,^[^ ^152^, ^153^ ^]^ failure to properly frame and analyze SDoH factors could limit the generalizability of these studies. In this perspective, we expanded the concept of GxE to the conceptual framework of GxSDoH, which may serve as a guide for researchers when conducting SDoH and genomics research. It is imperative to include SDoH factors in GxE‐COVID‐19 studies to better assess the role genes and the environment play by controlling for significant health determinants, such as behavioral, social and health system related SDoH. A summary of our recommendations for leaders, institutions, and researchers can be found in **Table** **2**.

**Table 2:** Summary of Recommendations for future research in GxE interactions and SDoH

Domain	Recommendation
Funding	Develop and maintain research and public health infrastructure to collect relevant data now, and not wait until the next crisis
	Responsive and timely funding mechanisms to support active inquiry
Methods	Establish standardized procedures and protocols for data collection, linkage, and analysis
	Implement data sharing, governance, and legal frameworks to support research
	Address missingness and variation in demographic and SDoH data sources
Community and education	Enhanced cross‐sector collaboration and promoting team science approaches
	Increase awareness of SDoH and their role in human health outcomes
	Enhance existing genetics resource with relevant SDoH data
Research topics	How to best capture, quantify and understand time of exposure in SDoH data
	Further elucidation of the mechanisms through which SDoH influence genetics and GxE

## Conflict of Interest

Dr. Ward‐Caviness is a paid scientific advisor for the Clock Foundation. The Clock Foundation had no role in this work. Juan Espinoza is a paid consultant for AI Health. AI Health had no role in this work. Nicole Weiskopf is a paid consultant for Merck. Merck had no role in this work.
